# Supplementary material for: Tunable translation-level CRISPR interference by dCas13 and engineered gRNA in bacteria
Source: Nat Commun. 2024 Jun 22;15:5319. doi: 10.1038/s41467-024-49642-x (PMC11193725; doi:10.1038/s41467-024-49642-x)
Supplement: Supplementary file 1 — Supplementary Information [file 41467_2024_49642_MOESM1_ESM.pdf]

Tunable translation-level CRISPR interference by dCas13 and engineered gRNA in bacteria

Kim *et al.*

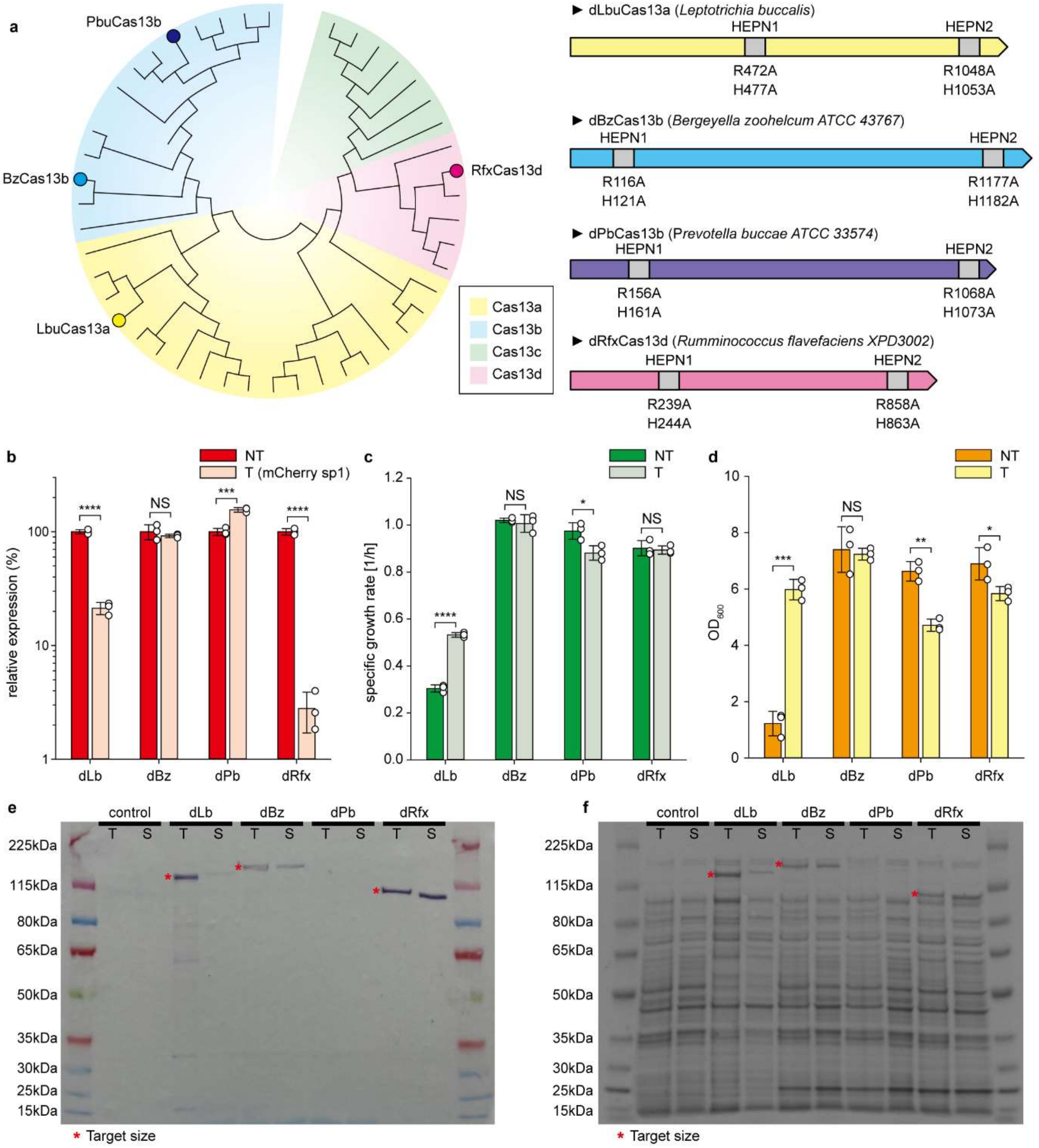

Supplementary Fig. 1. Comparison of four different dCas13 orthologs as a synthetic gene expression modulator for the Tl-CRISPRi system. a Phylogenetic tree of Cas13 orthologs and the selected Cas effector proteins (left). For the inactivation of non-specific nuclease activity of Cas13, the main catalytic residues of the HEPN domain (RxxxxH) were identified and substituted to the alanine (right), creating the dead Cas13 effector. The bacterial source of each Cas13 ortholog was denoted in the parenthesis. The guide RNA was expressed as the pre-crRNA form consisting of a repeat-spacer-repeat sequence. The reporter gene, *mCherry*, was constitutively expressed from the chromosome of *Escherichia coli* K-12 MG1655, and dCas13 orthologs were directed to target the 5` UTR of mCherry mRNA and block its translation. **b–d** The relative expression level of mCherry against the strain with the non-target guide RNA (**b**),

the specific growth rate of exponential phase (**c**), and the final cellular density (**d**) of bacterial strains were measured. NT: strain with non-target guide RNA, T: strain with guide RNA targeting *mCherry* (spacer: mCherry sp1). The error bar represents the mean ± standard deviation from the biologically independent cell cultures (n = 3), and the white dots indicate the actual data points. The *P*-value of each strain's dataset was determined by the two-tailed Student's *t*-test compared to the dataset of the NT strain. The asterisk indicates the *P*-value. NS: not significant; $*P < 0.05$, $**P < 0.01$, $***P < 0.001$, $****P < 0.0001$. **e**, **f** For the strains expressing *mCherry*-targeting guide RNA, the expression of dCas13 with N-terminal His-tag(6x) was analyzed by the western blot (**e**) and the coomassie blue staining (**f**), which was performed once for each experiment. The location of the full-length protein band was denoted by an asterisk (dLb-NHis: 140.3kDa, dBz-NHis: 147.6kDa, dPb-NHis: 135.6kDa, dRfx-NHis: 113.6kDa). control, parental strain without dCas13 expression; dLb, dLbuCas13a; dBz, dBzCas13b; dPb, dPbCas13b; dRfx, dRfxCas13d; T, total fraction; S, soluble fraction. Source data are provided as a Source Data file.

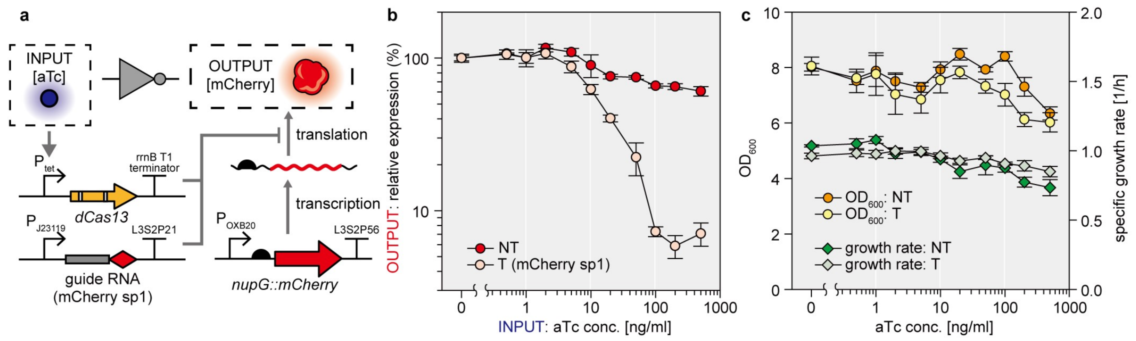

Supplementary Fig. 2. Input-responsive characteristics of the TI-CRISPRi system. a The *dCas13* gene was expressed under the aTc-inducible promoter (P_{tet}). **b** The relative expression level of mCherry was evaluated by normalizing the RFU/OD_{600} of mCherry without aTc as 100(%). **c** The optical density (OD_{600}) at the time point of measuring fluorescence and the specific growth rate of each strains during exponential growth phase. NT: strain with non-target guide RNA, T: strain with guide RNA targeting *mCherry* (spacer: mCherry sp1). The error bar represents the mean ± standard deviation from the biologically independent cell cultures (n = 3). Source data are provided as a Source Data file.

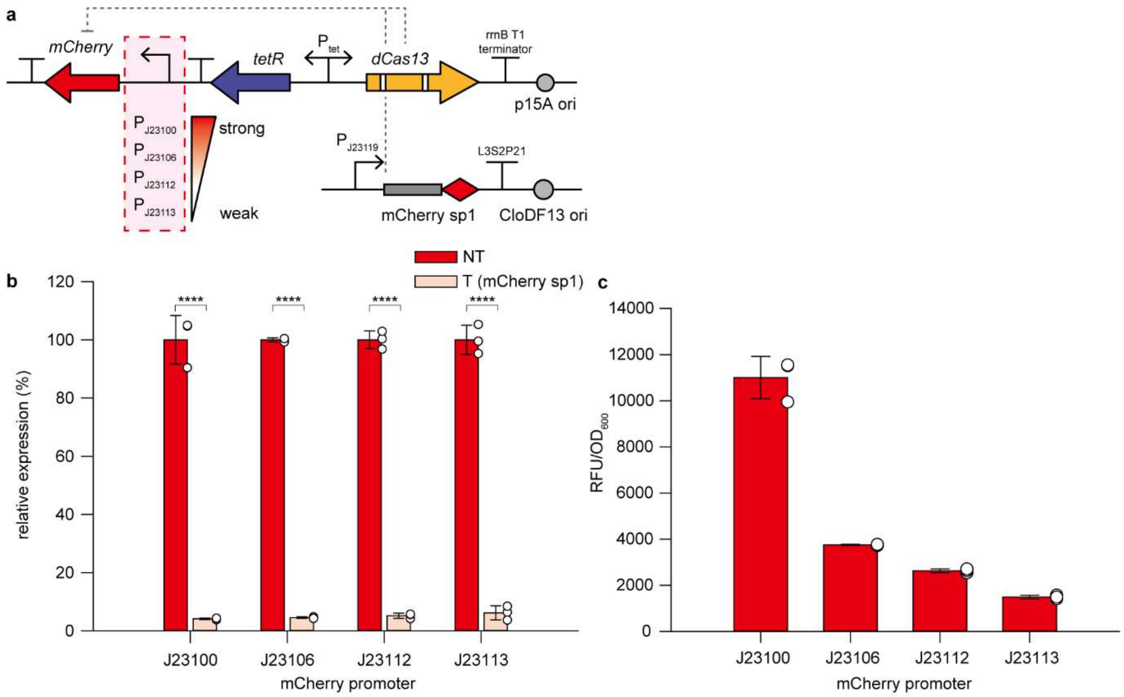

Supplementary Fig. 3. Knockdown of the mCherry mRNA transcribed from the plasmid upon various promoter strengths. a To differentiate the amount of the target transcript (mCherry mRNA), we implemented four different Anderson promoters (J23100, J23106, J23112, J23113) to derive the transcription of the *mCherry* gene. **b** The relative expression level of mCherry when the effective guide RNA (mCherry sp1) was adopted compared to the non-target guide RNA. NT: strain with non-target guide RNA, T: strain with guide RNA targeting *mCherry* (mCherry sp1). **c** Different expression rates of mCherry in the NT strains. The error bar represents the mean ± standard deviation from the biologically independent cell cultures (n = 3), and the white dots indicate the actual data points. The *P*-value of each strain's dataset was determined by the two-tailed Student's *t*-test compared to the dataset of the NT strain. The asterisk indicates the *P*-value. ****$P < 0.0001$. Source data are provided as a Source Data file.

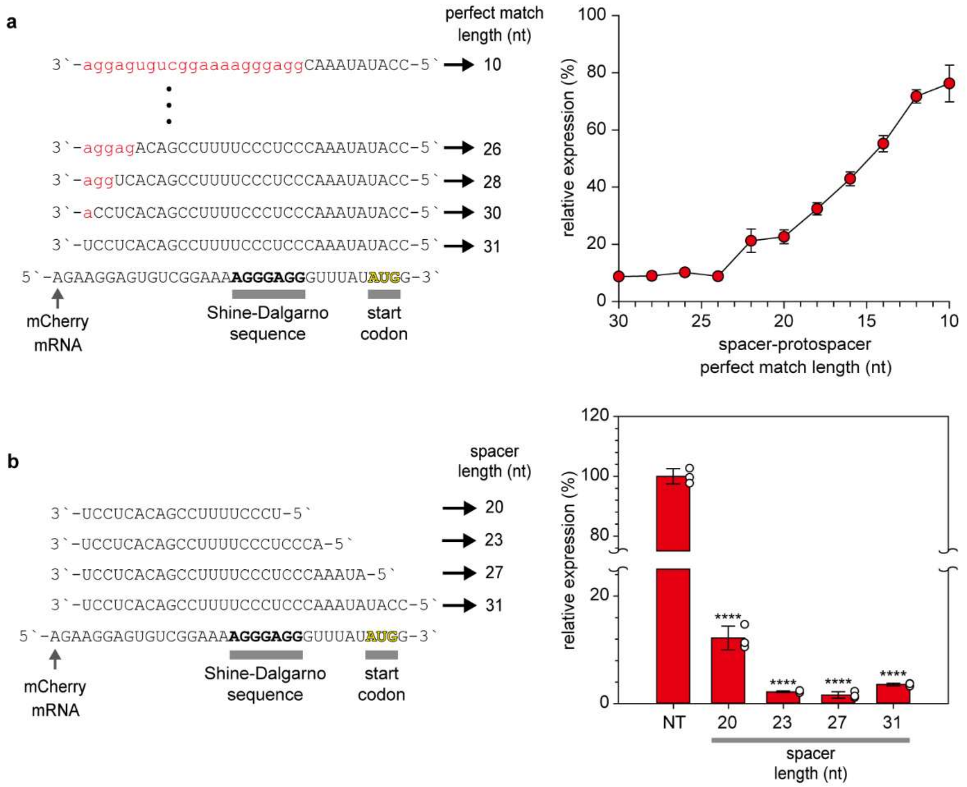

Supplementary Fig. 4. Varying the spacer-protospacer match length and the effect on knockdown efficiency. a While anchoring the 5` end of the spacer (mCherry sp1), mismatched bases were introduced from the 3` end, varying the length of complementary sequence between the spacer and the protospacer (5` UTR of mCherry mRNA). **b** Similarly, spacers with shortened lengths (27-nt, 23-nt, 20-nt) were adopted while maintaining the 3` end of the spacer. The relative expression level of mCherry was evaluated by normalizing the RFU/OD_{600} of mCherry of the NT (non-targeted) strain as 100(%). The error bar represents the mean ± standard deviation from the biologically independent cell cultures (n = 3), and the white dots indicate the actual data points. The *P*-value of each strain's dataset was determined by the two-tailed Student's *t*-test compared to the dataset of the NT strain. The asterisk indicates the *P*-value. ****$P < 0.0001$. Source data are provided as a Source Data file.

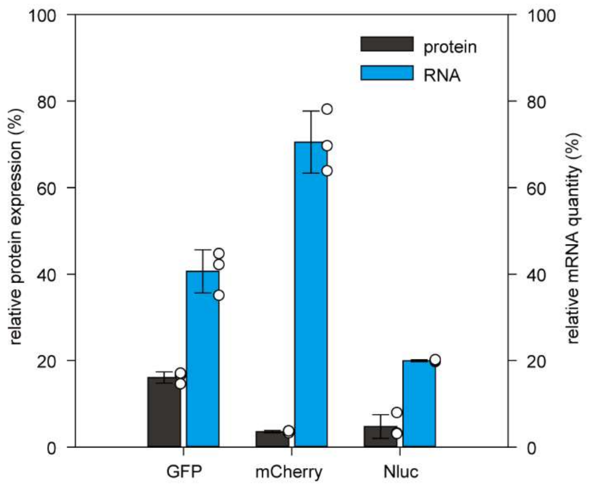

Supplementary Fig. 5. Relative reporter protein expression and mRNA quantity derived from the Tl-CRISPRi. For the knockdown of each reporter gene, the guide RNA with GFP sp1, mCherry sp1, or nanoluc sp1 was adopted, respectively. The relative expression level of the reporter protein was evaluated by normalizing the RFU or RLU/OD_{600} of the NT (non-targeted) strain as 100(%). The relative mRNA quantity was determined by RT-qPCR and the ddCt method, setting the transcript amount of NT strain as 100(%). The error bar represents the mean ± standard deviation from the biologically independent cell cultures (n = 3), and the white dots indicate the actual data points. Source data are provided as a Source Data file.

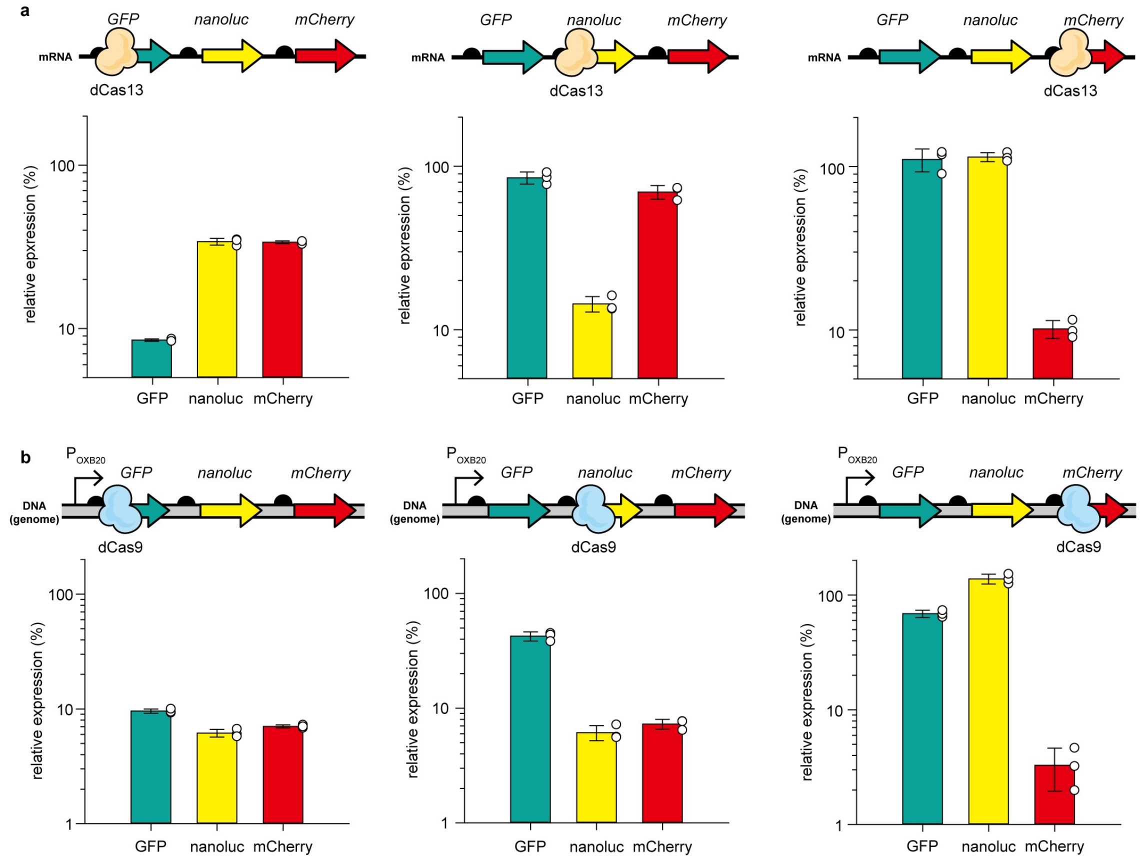

Supplementary Fig. 6. Comparing the Tx-CRISPRi and the Tl-CRISPRi on regulating the polycistronic gene expression (*GFP-mCherry-nanoluc*). The reporter gene operon consisting of *GFP*, *nanoluciferase*, and *mCherry* was expressed from the chromosome of *E. coli*. The guide RNA with the spacer mCherry sp1, GFP sp1, or nanoluc sp1 was adopted to knock down the expression of mCherry, GFP, or nanoluciferase (nanoluc), respectively. In comparison with the experiment of Figure 2, three reporter genes were arranged as *GFP*, *nanoluciferase*, and *mCherry* in series to construct the reporter gene operon. **a** Adopting Tl-CRISPRi toward the reporter gene operon. **b** Adopting Tx-CRISPRi toward the reporter gene operon. The relative expression level of the reporter protein was evaluated by normalizing the RFU or RLU/OD_{600} of the NT (non-targeted) strain as 100(%). The error bar represents the mean ± standard deviation from the biologically independent cell cultures (n = 3), and the white dots indicate the actual data points. Source data are provided as a Source Data file.

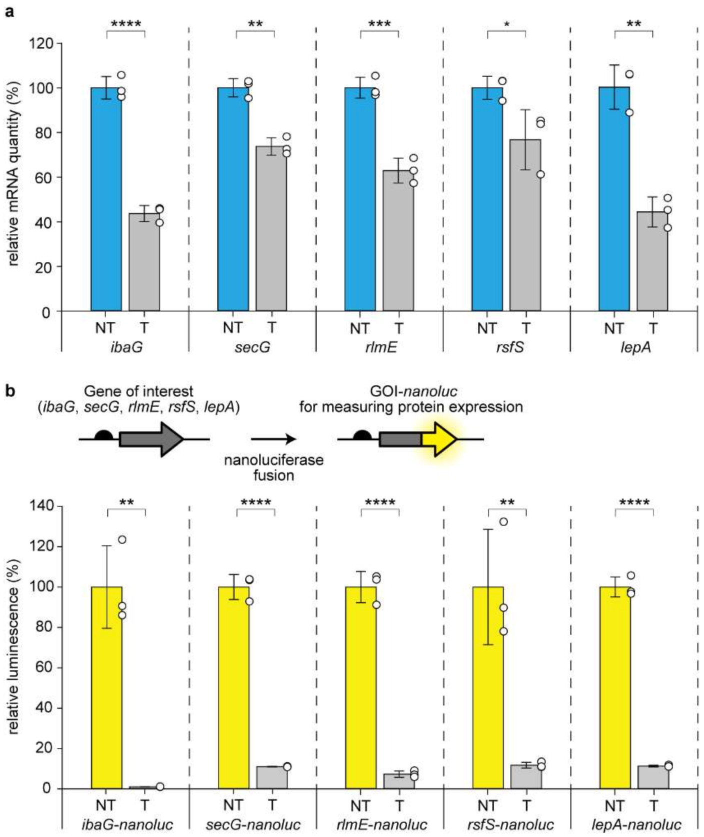

Supplementary Fig. 7. Perturbation on the RNA- and protein-level caused by the Tl-CRISPRi toward the endogenous genes in *E. coli*. a The mRNA level was quantified by RT-qPCR experiment to inspect whether the amount of targeted endogenous transcript was decreased by the Tl-CRISPRi. **b** To investigate whether the Tl-CRISPRi effectively knocked down the five endogenous genes targeted in the experiment of Figure 3, *nanoluciferase* (*nanoluc*) was fused to the C-terminal domain of each gene and the relative luminescence level of the strain with the effective guide RNA (T) was measured against that of the strain with non-target guide RNA (NT). Therefore, the relative luminescence level represents the decreased expression level of the gene targeted by Tl-CRISPRi. The error bar represents the mean ± standard deviation from the biologically independent cell cultures (n = 3), and the white dots indicate the actual data points. The P-value of each strain's dataset was determined by the two-tailed Student's t-test compared to the dataset of the NT strain. The asterisk indicates the P-value. *$P < 0.05$, **$P < 0.01$, ***$P < 0.001$, ****$P < 0.0001$. Source data are provided as a Source Data file.

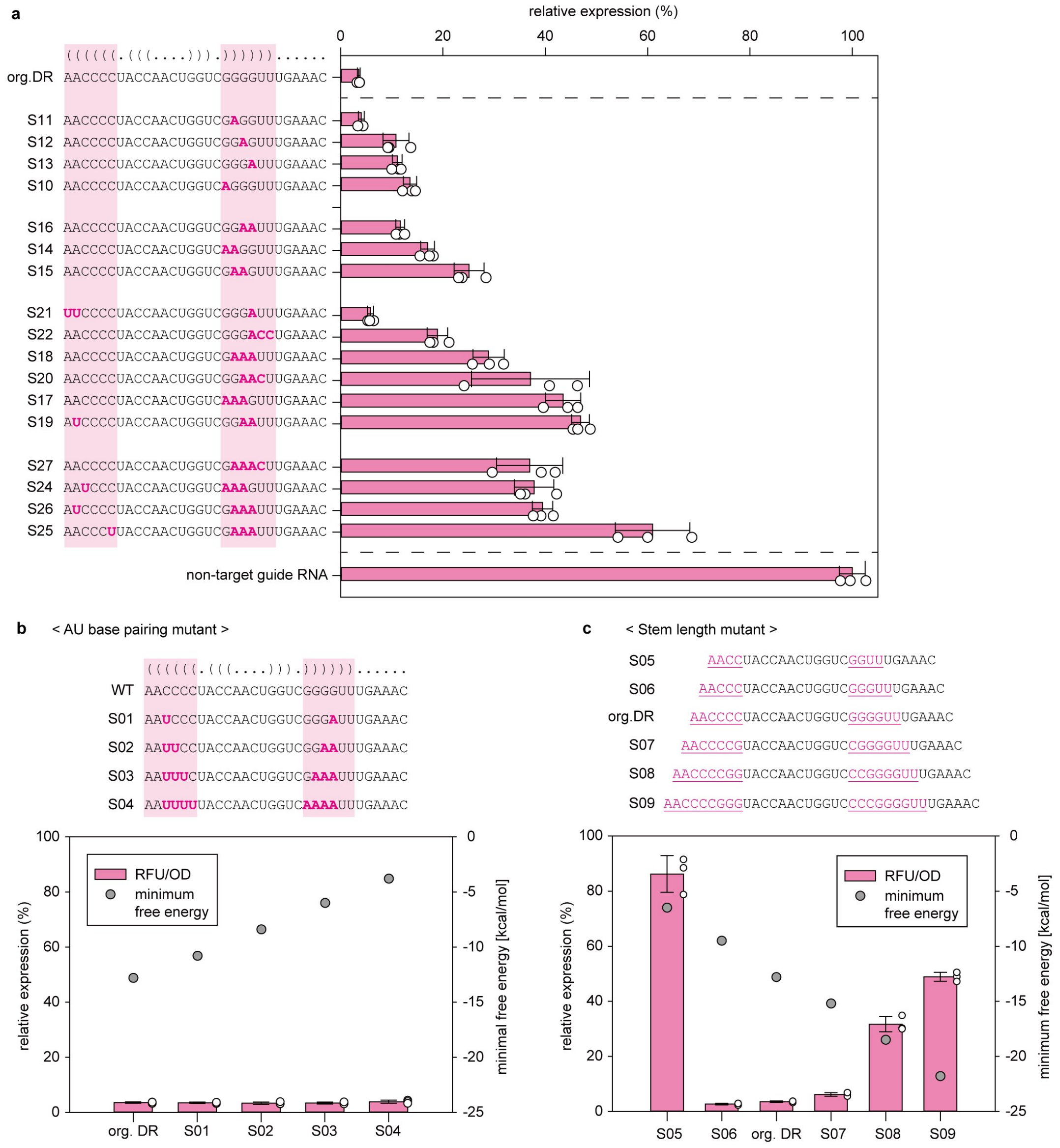

Supplementary Fig. 8. Relative expression level of mCherry derived from stem-mutated guide RNAs. a Disrupting the base pairing of the stem. **b** Replacing the GC pair into the AU pair. **c** Extending or shortening the stem length. The structure of the original DR (org. DR) was described based on the dot-parens-plus notation[1]. Bolded nucleotides (**a**, **b**) are mutations against the original DR sequence. Pink-colored shade (**a**, **b**) represents the 6-bp stem. Pink-colored, underlined letters (**c**) represent the nucleotides comprising length-varied stem. The minimum free energy (**b**, **c**) of the modified direct repeat was calculated based on the NUPACK software[1]. The relative expression level of mCherry was evaluated by normalizing the RFU/OD_{600} of mCherry of the NT (non-targeted) strain as 100(%). The error bar represents the mean ± standard deviation from the biologically independent cell cultures (n = 3), and the white dots indicate the actual data points. Source data are provided as a Source Data file.

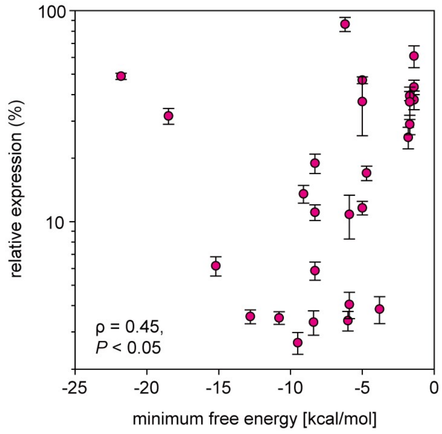

Supplementary Fig. 9. Comparison of relative expression level of mCherry derived from stem-mutated guide RNAs and minimum free energy of each stem-mutated DR. The minimum free energy of the modified direct repeat was calculated based on the NUPACK software[1]. The error bar represents the mean ± standard deviation from the biologically independent cell cultures (n = 3). Source data are provided as a Source Data file.

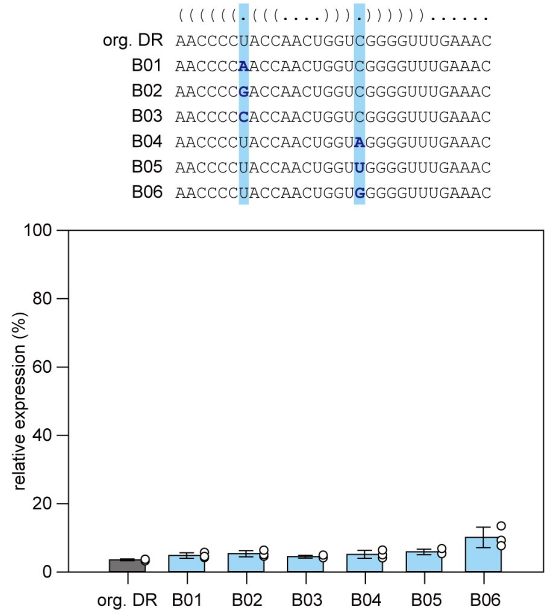

Supplementary Fig. 10. Relative expression level of mCherry derived from bulge-mutated guide RNAs. The structure of the original DR (org. DR) was described based on the dot-parens-plus notation[1]. Blue-colored shade represents the bulge nucleotides, and introduced mutations of each DR variant are described in bold letters. The relative expression level of mCherry was evaluated by normalizing the RFU/OD_{600} of mCherry of the NT (non-targeted) strain as 100(%). The error bar represents the mean ± standard deviation from the biologically independent cell cultures (n = 3), and the white dots indicate the actual data points. Source data are provided as a Source Data file.

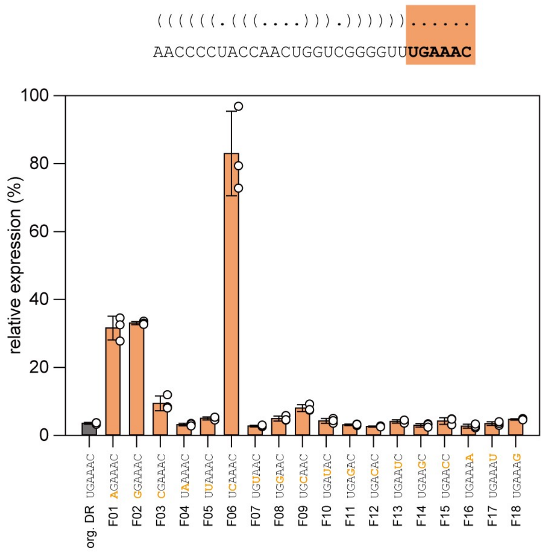

Supplementary Fig. 11. Relative expression level of mCherry derived from flanking sequence-mutated guide RNAs. The structure of the original DR was described based on the dot-parens-plus notation[1], which is above the graph. Orange-colored shade represents the nucleotides comprising the flanking sequence, and introduced mutations of each DR variant are described in orange-colored letters. The relative expression level of mCherry was evaluated by normalizing the RFU/OD_{600} of mCherry of the NT (non-targeted) strain as 100(%). The error bar represents the mean ± standard deviation from the biologically independent cell cultures (n = 3), and the white dots indicate the actual data points. Source data are provided as a Source Data file.

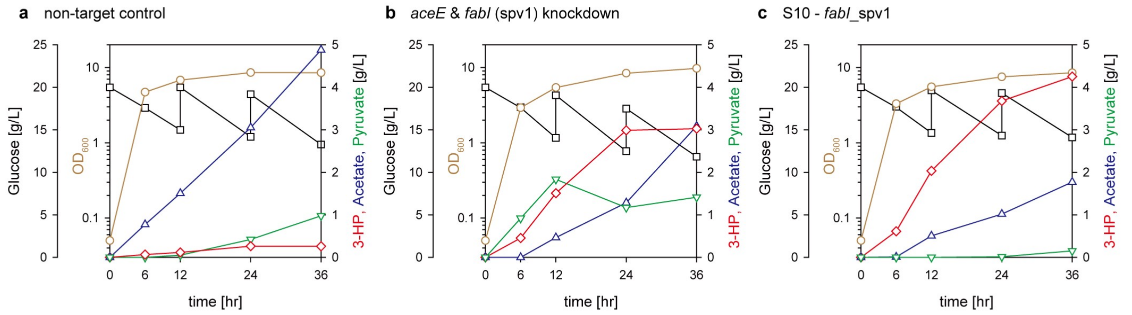

Supplementary Fig. 12. Fed-batch fermentation profile of strains under the Tl-CRISPRi regulation. **a** the strain with non-target guide RNA. **b** the strain with two guide RNAs each targeting *aceE* and *fabI*. **c** the strain repressing *fabI* with the mutated guide RNA (S10-*fabI* spv1). Circle, OD_{600}; rectangle, glucose; diamond, 3-HP; triangle, acetate; reverse-triangle, pyruvate. Source data are provided as a Source Data file.

Supplemenetary references

1. Zadeh, J. N. *et al.* NUPACK: Analysis and design of nucleic acid systems. *J. Comput. Chem.* **32**, 170–173 (2011).
