# Supplementary material for: Tunable translation-level CRISPR interference by dCas13 and engineered gRNA in bacteria
Source: Nat Commun. 2024 Jun 22;15:5319. doi: 10.1038/s41467-024-49642-x (PMC11193725; doi:10.1038/s41467-024-49642-x)
Supplement: Supplementary file 3 — Description of Additional Supplementary Files [file 41467_2024_49642_MOESM3_ESM.pdf]

Description of Additional Supplementary Files

File Name: Supplementary Data 1
Description: Strains and plasmids used in this study

File Name: Supplementary Data 2
Description: Primers used in this study

File Name: Supplementary Data 3
Description: Spacers and DR sequences used in this study
