# Supplementary material for: Tunable translation-level CRISPR interference by dCas13 and engineered gRNA in bacteria
Source: Nat Commun. 2024 Jun 22;15:5319. doi: 10.1038/s41467-024-49642-x (PMC11193725; doi:10.1038/s41467-024-49642-x)
Supplement: Supplementary file 7 — Reporting Summary [file 41467_2024_49642_MOESM7_ESM.pdf]

nature portfolio

Corresponding author(s): Sang Woo Seo

Last updated by author(s): Jun 5, 2024

Reporting Summary

Nature Portfolio wishes to improve the reproducibility of the work that we publish. This form provides structure for consistency and transparency in reporting. For further information on Nature Portfolio policies, see our Editorial Policies and the Editorial Policy Checklist.

Statistics

For all statistical analyses, confirm that the following items are present in the figure legend, table legend, main text, or Methods section.

n/a	Confirmed	
☐	☒	The exact sample size (*n*) for each experimental group/condition, given as a discrete number and unit of measurement
☐	☒	A statement on whether measurements were taken from distinct samples or whether the same sample was measured repeatedly
☐	☒	The statistical test(s) used AND whether they are one- or two-sided<br>*Only common tests should be described solely by name; describe more complex techniques in the Methods section.*
☒	☐	A description of all covariates tested
☒	☐	A description of any assumptions or corrections, such as tests of normality and adjustment for multiple comparisons
☐	☒	A full description of the statistical parameters including central tendency (e.g. means) or other basic estimates (e.g. regression coefficient) AND variation (e.g. standard deviation) or associated estimates of uncertainty (e.g. confidence intervals)
☐	☒	For null hypothesis testing, the test statistic (e.g. *F*, *t*, *r*) with confidence intervals, effect sizes, degrees of freedom and *P* value noted<br>*Give P values as exact values whenever suitable.*
☒	☐	For Bayesian analysis, information on the choice of priors and Markov chain Monte Carlo settings
☒	☐	For hierarchical and complex designs, identification of the appropriate level for tests and full reporting of outcomes
☐	☒	Estimates of effect sizes (e.g. Cohen's *d*, Pearson's *r*), indicating how they were calculated

Our web collection on statistics for biologists contains articles on many of the points above.

Software and code

Policy information about availability of computer code

Data collection	For fluorescence and luminescence measurement, PlateReaderSoftware v0.5.27.0 (Hidex) was used for data collection.<br>RT-qPCR data were collected using StepOne Software v2.3 (Applied Biosystems).<br>Openlab CDS v2.15.26 (Agilent) was used for HPLC data collection.
Data analysis	For the generation of the phylogenetic tree of Cas13 orthologs, MEGA v11.0.10 software was used.

For manuscripts utilizing custom algorithms or software that are central to the research but not yet described in published literature, software must be made available to editors and reviewers. We strongly encourage code deposition in a community repository (e.g. GitHub). See the Nature Portfolio guidelines for submitting code & software for further information.

Data

Policy information about availability of data

All manuscripts must include a data availability statement. This statement should provide the following information, where applicable:

- Accession codes, unique identifiers, or web links for publicly available datasets
- A description of any restrictions on data availability
- For clinical datasets or third party data, please ensure that the statement adheres to our policy

All data supporting the findings of this work are available within the paper and its Supplementary Information file. Bacterial strains and plasmids used in this study,

and the other datasets generated and analyzed during this study are available from the corresponding author upon request. Source data are provided with this paper.

Research involving human participants, their data, or biological material

Policy information about studies with human participants or human data. See also policy information about sex, gender (identity/presentation), and sexual orientation and race, ethnicity and racism.

Reporting on sex and gender	Not applicable
Reporting on race, ethnicity, or other socially relevant groupings	Not applicable
Population characteristics	Not applicable
Recruitment	Not applicable
Ethics oversight	Not applicable

Note that full information on the approval of the study protocol must also be provided in the manuscript.

Field-specific reporting

Please select the one below that is the best fit for your research. If you are not sure, read the appropriate sections before making your selection.

☒ Life sciences ☐ Behavioural & social sciences ☐ Ecological, evolutionary & environmental sciences

For a reference copy of the document with all sections, see nature.com/documents/nr-reporting-summary-flat.pdf

Life sciences study design

All studies must disclose on these points even when the disclosure is negative.

Sample size	Most of the experiments were performed in triplicates, which normally ensures biological reproducibility in the bacterial cell culture. The bacterial culture and HPLC analysis presented in Figure 5b were conducted in duplicates as this experiment was aimed to swiftly find out effective gene knockdown targets among about 48 different genes and choose gene candidates for the following experiments, which were then performed in triplicates. The fed-batch cultures in Supplementary Figure 12 were conducted by single biological sample as this experiment was the repetition of the experiment in Figure 5 using the identical strain while scaling up the culture volume.
Data exclusions	No data were excluded.
Replication	All attempts for fluorescence and luminescence measurement, RT-qPCR, and metabolite quantification using HPLC were successfully performed in biologically independent duplicates or triplicates. We validated that our findings were reproducible by conducting experiments on the different days. The number of replicates was noted in the manuscript.
Randomization	This is not relevant since we did not generated randomized libraries and tested same genotype microorganisms for each experiment.
Blinding	Blinding is not relevant with this study, since all experiments we conducted here were quantitative and evident. Computational software was used for fluorescence and luminescence analysis and metabolite quantification equally to all attempts and replicates for a given condition.

Reporting for specific materials, systems and methods

We require information from authors about some types of materials, experimental systems and methods used in many studies. Here, indicate whether each material, system or method listed is relevant to your study. If you are not sure if a list item applies to your research, read the appropriate section before selecting a response.

Materials & experimental systems

n/a	Involved in the study
☐	☒ Antibodies
☒	☐ Eukaryotic cell lines
☒	☐ Palaeontology and archaeology
☒	☐ Animals and other organisms
☒	☐ Clinical data
☒	☐ Dual use research of concern
☒	☐ Plants

Methods

n/a	Involved in the study
☒	☐ ChIP-seq
☒	☐ Flow cytometry
☒	☐ MRI-based neuroimaging

Antibodies

Antibodies used

6x-His Tag Monoclonal Antibody (HIS.H8), eBioscience™ (cat.number 14-6657-82) was provided by eBioscience. Anti-Mouse IgG (whole molecule)−Alkaline Phosphatase antibody produced in goat (cat.number A9316) was provided by Sigma. The 6x-His Tag Monoclonal Antibody was diluted to 3:10000, and the Anti-Mouse IgG (whole molecule)-Alkaline Phosphatase antibody was diluted to 1:10000 during the antibody incubation steps.

Validation

These antibodies are commercially validated and available. (anti-His, https://www.thermofisher.com/antibody/product/6x-His-Tag-Antibody-clone-HIS-H8-Monoclonal/14-6657-82; anti-mouse, https://www.sigmaaldrich.com/KR/ko/product/sigma/a9316) We checked that no band was detected in the western blotting of wild-type cell, which was shown in Supplementary Figure 1e.

Plants

Seed stocks

Not applicable

Novel plant genotypes

Not applicable

Authentication

Not applicable
